# Tumour associated endothelial cells: origin, characteristics and role in metastasis and anti-angiogenic resistance

**DOI:** 10.3389/fphys.2023.1199225

**Published:** 2023-06-14

**Authors:** Xinghong Yao, Ye Zeng

**Affiliations:** ^1^ Radiation Oncology Key Laboratory of Sichuan Province, Department of Radiotherapy, Sichuan Clinical Research Center for Cancer, Sichuan Cancer Hospital and Institute, Sichuan Cancer Center, Affiliated Cancer Hospital of University of Electronic Science and Technology of China, Chengdu, China; ^2^ Institute of Biomedical Engineering, West China School of Basic Medical Sciences and Forensic Medicine, Sichuan University, Chengdu, China

**Keywords:** tumour, tumour-associated endothelial cells, characteristics, metastasis, anti-angiogenic therapy

## Abstract

Tumour progression and metastasis remain the leading causes of cancer-related death worldwide. Tumour angiogenesis is essential for tumour progression. The vasculature surrounding tumours is not only a transport channel for nutrients, oxygen, and metabolites, but also a pathway for metastasis. There is a close interaction between tumour cells and endothelial cells in the tumour microenvironment. Recent studies have shown that tumour-associated endothelial cells have different characteristics from normal vascular endothelial cells, play an important role in tumour progression and metastasis, and are expected to be a key target for cancer therapy. This article reviews the tissue and cellular origin of tumour-associated endothelial cells and analyses the characteristics of tumour-associated endothelial cells. Finally, it summarises the role of tumour-associated endothelial cells in tumour progression and metastasis and the prospects for their use in clinical anti-angiogenic therapy.

## 1 Introduction

Angiogenesis was introduced by British surgeon John Hunter over 200 years ago to describe the growth of blood vessels in reindeer antlers (Ren et al., 2019). In 1971, Judah Folkman proposed that tumour growth is entirely dependent on angiogenesis (Carmeliet and Jain, 2000).

Angiogenesis is essential for tumour progression. Without blood vessels, tumours remain a mass of cells less than 2 mm in diameter (Ribatti, 2008). The blood vessels surrounding the tumour not only supply nutrients, oxygen and metabolites, but also provide a pathway for tumour cells to metastasise. Therefore, angiogenesis has become a key target for cancer therapy, and several anti-angiogenic drugs/inhibitors have been developed. However, anti-angiogenic therapy is a double-edged sword, with the positive benefits of anti-angiogenic drugs such as reduction of tumour size and prolonged the survival as giving hope to patients and the negative side effects such as hypertension, proteinuria, bleeding, thrombosis and hypothyroidism leaving clinicians conflicted (Li et al., 2022; Motzer et al., 2022; Lee N. et al., 2023). A major dilemma facing clinical anti-angiogenic therapy today is how to maximise treatment efficacy and minimise side effects.

The success or failure of the tumour metastatic cascade response is highly dependent on the interactions between components of the tumour and the local microenvironment through a variety of bioactive molecules such as growth factors, cytokines, chemokines, and microRNAs (Sellner et al., 2022). A particular site of intercellular communication within the tumour is the vasculature. Within the vasculature, tumour cells come into direct contact with tumour-associated endothelial cells (TECs). Tumour-endothelial interactions not only promote malignant vascularisation but also influence the proliferative and metastatic potential of the malignant cells (Hashemi et al., 2022).

VEGF-A (referred to as VEGF) is the major angiogenesis-inducing molecule released by tumour cells (Qi et al., 2023). Excess VEGF in tumours can lead to excessive vascular permeability and increased inter-tissue osmotic pressure, resulting in disorganised vascular architecture. The tumour vasculature is complex, unstratified and disorganized (Balan et al., 2019). In turn, TECs can release vascular secretory factors that promote tumour progression (Butler et al., 2010). TECs can secrete biglycan, which facilitates the metastasis (Maishi et al., 2016).

TECs are found on the inner surface of tumour vessels and are previously considered to be genetically stable (Hillen and Griffioen, 2007). In fact, TECs are distinct from normal vascular endothelial cells and their properties can be exploited in translational research for cancer intervention and treatment. Currently, tumour-derived primary endothelial cells have emerged as a more representative way to assess tumour angiogenesis (Liu et al., 2020). This review focuses on an overview of the origin, features, and role of TECs in tumour progression and metastasis, and their potential application in clinical anti-angiogenic therapy.

## 2 Origin of TECs

### 2.1 Tumour blood vessels

Tumor blood vessels are the major source of TECs. TECs can be isolated using the endothelial markers, but they possess some specific characteristics, including the absence of von Willebrand factor (vWF), which distinguishes them from normal vascular endothelial cells (Naschberger et al., 2011).

TECs including tumour-derived vascular endothelial cells (TDVEC) and tumour-derived lymphatic endothelial cells (TDLEC) have been identified in tumours. TEVEC isolated from malignant gliomas express an enriched angiogenesis-related gene profile, whereas TDLEC express interferon-alpha and interferon-beta, which may be involved in inflammation and immune cell recruitment (Carlson et al., 2021). Comparison of a mouse model of endogenous glioma with a patient derived orthotopic xenograft (PDOX) model showed that tumour-associated vascular endothelium is distinct from normal brain endothelium and exhibits extensive heterogeneity (Carlson et al., 2021).

Delivery of extracellular vesicles or non-coding RNAs into the tumour microenvironment plays an essential role in tumour angiogenesis (Li et al., 2021). After overexpression of miR-9, vascular endothelial cells can possess enhanced angiogenic ability, and the generated exosomes followed anti-angiogenic therapy can in turn promote the vascular mimicry of tumour cells and the progression of the abnormal tumour vasculature (Zeng et al., 2019). The extracellular vesicles from the lung cancer cell line A549 cells can induce abnormal angiogenesis by downregulating transient receptor potential vanilloid 4 (TRPV4) and activating Rho/Rho kinase/YAP/VEGFR2 pathways (Guarino et al., 2021). The mechanisms by which the tumour microenvironment modifies endothelial cells are still poorly understood.

### 2.2 Tumour stem cells

Tumour stem cells may be involved in angiogenesis to form tumour vasculature. Cells derived from tumour to endothelial transdifferentiation express typical endothelial marker CD31 and vWF and stem cell markers such as Nestin and CD133, and cells contributed to vasculogenic mimicry lack CD34 and CD31 expression (He et al., 2012; Maddison et al., 2023).

Glioblastoma endothelial cells have a similar expression profile to other tumour cells. Glioblastoma stem cells positive for the stem cell marker CD133 may have an endothelial-like phenotype and function (Ricci-Vitiani et al., 2010; Wang et al., 2010). However, it has been reported that TDVEC are rarely found in glioblastoma vasculature (Rodriguez et al., 2012). Nonetheless, glioma stem cells can differentiate into perivascular cells and thus support tumour angiogenesis and progression (Cheng et al., 2013). The use of a combinatorial treatment of RoboN and the monoclonal anti-SDF-1 antibody effectively attenuated the activity of hemangioblast derived-endothelial cell precursors and tumor neovascularization (Shenoy et al., 2023). CD44/CD44v6 might play a vital role in formation of cancer stem cells and angiogenesis (Wang et al., 2018).

Although transdifferentiation of tumor stem cells to enodothelial cells have been suggested in vasculogenic mimicry, whether tumour stem cells are origin of TECs and the transformation mechanism remains unclear.

### 2.3 Endothelial progenitor cells

Endothelial progenitor cells recruited from bone marrow have the capacity to differentiate into mature vascular endothelial cells (Zhou et al., 2013). Endothelial CD34^+^CD45– progenitors formed entire blood vessels in wounds, inflamed skin, and tumors (Patel et al., 2017). The CD34 were progressively lose in endothelial progenitor cells during cultivation and maturation (Delia et al., 1993).

CD133+ bone marrow cells, a subset of CD34^+^ progenitors, can retain high levels of VEGFR2 expression while positively expressing VE-cadherin, vWF, and platelet endothelial cell adhesion molecule 1 (PECAM1, CD31) (Quirici et al., 2001). Another study suggested that CD133+ cells were unable to differentiate into mature endothelial cells, whereas CD34^+^ endothelial progenitors could (Lu et al., 2007). It is possible that CD133+/CD34+ endothelial progenitor cells are a stable origin of TECs. However, the specific mechanism by which the endothelial progenitor cells differentiate into TECs is not yet known.

## 3 Characteristics of TECs

### 3.1 Genetic instability

TECs from xenografts, such as human melanoma and liposarcoma, have larger nuclei than normal vascular endothelial cells from skin and adipose, and cytogenetic abnormalities (Hida et al., 2004). Lymphatic-specific chromosomal translocations have also been found in microvascular endothelial cells of B-cell lymphomas (Streubel et al., 2004). In addition to tumour-resident TECs, aneuploid CD31^+^ circulating TECs are found in the peripheral blood of breast cancer patients (Lin et al., 2017). TECs isolated from xenografted human epithelial tumours are CD133+ cells, have a higher frequency of aneuploidy than CD133-cells and are genetically unstable (Akino et al., 2009).

Hypoxia-induced reactive oxygen species and VEGF signalling promote chromosomal abnormalities of TECs (Kondoh et al., 2013). Vascular endothelial cells may receive tumour genetic material (DNA/chromosomes) delivered by tumour cell-derived apoptotic vesicles (Bergsmedh et al., 2001; Ehnfors et al., 2009), resulting in altered cytogenetics.

Cell fusion between transformed cells and normal stem cells may be important in the genomic transfer and generation of reprogrammed somatic cell hybrids with self-renewing highly malignant phenotype (Glinsky, 2005). The formation of cell-in-cell structures (a type of unique structure with one or more cells within another one) may promote genetic transfer and contribute to genomic instability and malignancy of tumor cells (Wang et al., 2023). Thus, fusion of bone marrow-derived endothelial progenitor cells with TECs might be responsible for genetic instability.

### 3.2 Genetic heterogeneity

TECs are composed of heterogeneous cell populations (Hennigs et al., 2021). Single-cell RNA sequencing has also demonstrated the heterogeneity of TECs in mouse and human samples (Goveia et al., 2020). The heterogeneity of TECs is influenced by tumour type, extracellular environment and epigenetic regulation (Aird, 2009). TECs have distinct and heterogeneous gene expression patterns compared to normal vascular endothelial cells (Hennigs et al., 2021). Genes involved in the regulation of angiogenesis, cell proliferation and motility, stemness and drug resistance are differentially upregulated in TECs (Ohga et al., 2012). TECs express high levels of VEGFR1 and VEGFR2, which enhances cellular responsiveness to VEGF and angiogenesis (Matsuda et al., 2010). They have the capacity for survival in serum-free medium (Bussolati et al., 2003). Compared to normal colorectal mucosal endothelium, 46 genes were specifically elevated in endothelial cells isolated from colorectal cancer, including MMP2 and MMP11, and collagen types I, III and VI (St Croix et al., 2000). A map of single-cell sequencing in hepatocellular carcinoma (HCC) shows that in TECs, extracellular matrix organization genes such as COL4A2 and SPARC were upregulated, liver sinusoidal endothelial cell (LSEC) markers such as CLEC4G did not express, while macro-vascular endothelial cell (MVEC) markers such as PECAM1 (CD31), AQP1 and CD34 were also upregulated (Aizarani et al., 2019). Furthermore, HCC LSECs express plasmalemma vesicle-associated protein (PLVAP), making them less permeable and potentially limiting the entry of lymphocytes and tumour-derived antigens (Rantakari et al., 2015). PLVAP is considered as a major factor influencing the permeability of endothelial cells, also upregulated within pro-angiogenic or pro-inflammatory responses (Denzer et al., 2023).

### 3.3 Anoikis resistance

Anoikis (Anchorage-Dependent Cell Death) refers to the death of normal adnexal cells that are left suspended for long periods and become ‘homeless’. Non-coding RNAs are closely related to anoikis resistance of cancer cells (Shi et al., 2022). TECs with high expression of miR-145 have an increased ability to resist anoikis (Hida et al., 2017). Circulating TECs with high expression of Bcl-2 also have increased resistance to anoikis (Yadav et al., 2015). The increased resistance of TECs to anoikis enhances their ability to survive after entry into the circulation, while improving cell adhesion and promoting tumour metastasis (Yadav et al., 2015). TECs interacting with tumour cells can protect the latter from anoikis in circulation and help them to migrate to distant organs (Pyaskovskaya et al., 2021). Silencing of syndecan-4 led to an inhibition of the proliferative, invasive and angiogenic capacity of anoikis-resistant endothelial cells (Onyeisi et al., 2020).

### 3.4 Abnormal metabolism

Angiogenesis of lung TEC is closely associated with glycolysis and oxidative stress (Wu et al., 2018). Inhibition of glycolysis tightened the vascular barrier and inhibited the expression of cancer cell adhesion molecules in endothelial cells (Cantelmo et al., 2016). Pharmacological blockade of cyclooxygenase-2 (COX-2) by parixibox restored the glucose metabolism level (particularly glycolysis) in TECs, reducing the VEGF (Zhang et al., 2018). Inhibition of the glycolytic activator PFKFB3 tightened the vascular barrier by reducing VE-cadherin endocytosis in TECs, contributing to the reduction of metastasis by normalizing tumor vessels (Cantelmo et al., 2016). Deficiency of endothelial TRPM7 inhibited postnatal retinal angiogenesis in mice might through normalized glycolytic metabolism (Wu et al., 2023). Lactate activated the major stroma components in gastric cancer mesenchymal stem cells and promote their migration (Tao et al., 2023). Unlike normal endothelial cells, TECs proliferate in lactic acidic via upregulation of carbonic anhydrase 2 (CA2) (Annan et al., 2019). The mechanism by which TECs survive in a high-lactate environment and participate in angiogenesis is required to be further explored.

### 3.5 Resistance to anticancer drugs

Following first-line chemotherapy, the proportion of ABCB1-postive TEC in urothelial carcinoma was increased, which conferred drug resistance to the tumor (Kikuchi et al., 2020).

TECs isolated from highly metastatic human melanoma cell xenografts express high levels of multidrug resistance protein 1 (MDR1) and are resistant to the anticancer drug paclitaxel (Akiyama et al., 2012). CD105+ TECs isolated from hepatocellular carcinoma were resistant to doxorubicin and 5-fluorouracil (Xiong et al., 2009). The PD-L1+ aneuploid circulating TECs exhibit resistance to the checkpoint blockade immunotherapy in advanced non-small cell lung cancer patients (Zhang et al., 2020).

TECs highly express stemness markers such as CD105 and CD146 (St Croix et al., 2000; Otsubo et al., 2014). The stem cell properties of TECs may be responsible for the development of drug resistance, but further clarification is required.

### 3.6 Adaptive to abnormal mechanical microenvironment

Tumour-associated vasculature is characterised by irregular morphology and structure, dysfunction, and reduced flow and leakage in comparison to normal vessels. The stiffness of tumour-derived extracellular matrix is increased, leading to high interstitial pressure (Paszek et al., 2005). Increased interstitial pressure and reduced blood flow within the tumour vasculature reduces shear stress and inhibits leukocyte rolling but promote their adhesion to the endothelium (Finger et al., 1996). Endothelial cells can sense the stiffness of the extracellular matrix. In contrast to a soft stroma, a hard stroma led to dysfunction of the endothelial monolayer, independently from inflammatory signalling (Onken et al., 2014). The composition of extracellular matrix such as geltrex and collagen I is critical for endothelial cell function (Riddle et al., 2022). It remains unknown whether the mechanical responsiveness of TECs differs from that of normal vascular endothelial cells.

The major characteristics of TEC are summarized in Table 1.

**TABLE 1:** Characteristics of TECs.

Features	Manifestations	Tumour/signalling	Ref
Genetic instability	Larger nuclei	Human melanoma and liposarcoma	Hida et al. (2004)
	Chromosomal translocations	B-cell lymphomas	Streubel et al. (2004)
	Aneuploid	Breast cancer	Lin et al. (2017)
	Genomic transfer	Cell fusion/cell-in-cell structure	Glinsky (2005), Wang et al. (2023)
Genetic heterogeneity	Heterogeneous gene expression patterns	Influenced by tumour type, extracellular environment, and epigenetic regulation	Aird (2009), Hennigs et al. (2021)
	Upregulated genes involved in angiogenesis (VEGFR1/2), cell proliferation and motility, stemness and drug resistance		Matsuda et al. (2010), Ohga et al. (2012)
	Survival capacity in serum-free medium		Bussolati et al. (2003)
	Upregulated extracellular matrix organisation genes such as COL4A2 and SPARC, and vascular endothelial cell markers such as CD31, AQP1 and CD34	Hepatocellular carcinoma	Aizarani et al. (2019)
Anoikis resistance	Resistance to anoikis	miR-145, Bcl-2, syndecan-4	Yadav et al. (2015), Hida et al. (2017), Onyeisi et al. (2020)
	Protected tumour cells from anoikis in circulation and helped them to migrate to distant organs		Pyaskovskaya et al. (2021)
Abnormal metabolism	Closely associated with glycolysis and oxidative stress	COX-2, PFKFB3, TRPM7, CA2	Cantelmo et al. (2016), Wu et al. (2018), Zhang et al. (2018), Annan et al. (2019), Wu et al. (2023)
Resistance to anticancer drugs	ABCB1-postive TEC increased after first-line chemotherapy	Urothelial carcinoma	Kikuchi et al. (2020)
	Resistant to paclitaxel	Human melanoma cell xenografts, MDR1	Akiyama et al. (2012)
	Resistant to doxorubicin and 5-fluorouracil	Hepatocellular carcinoma, CD105	Xiong et al. (2009)
	Resistant to the checkpoint blockade immunotherapy	Advanced non-small cell lung cancer, PD-L1	Zhang et al. (2020)
Adaptive to abnormal mechanical microenvironment	Inhibited leukocyte rolling but promoted their adhesion	Increased extracellular matrix stiffness; increased interstitial pressure and reduced blood flow/shear stress	Finger et al. (1996), Paszek et al. (2005)
	Specific mechanical responsiveness	Independent of Inflammatory signalling; dependent on extracellular matrix composition	Onken et al. (2014), Riddle et al. (2022)

## 4 TECs in tumour progression and metastasis

Tumour blood vessels, with abnormal thin-wall, contribute to metastasis (Oka et al., 2023). The immature nature of tumour vessels, with the absence of smooth muscle cells or pericytes, allows tumour cells to readily invade tumour vessels (Morikawa et al., 2002).

TECs may also provide signals that actively promote metastasis. CXCR7 regulated CXCL12-mediated migratory cues, driving CXCR4+CXCR7+ metastasis and tissue invasion (Zabel et al., 2009). However, in hepatocellular carcinoma patients, CXCR4 affected overall survival, but not CXCR7 (Neve Polimeno et al., 2015). Conditionally deleted the CXCR7 from vascular endothelium of adult mice elevated number of circulating tumor cells and more spontaneous metastases (Stacer et al., 2016). Transendothelial migration of tumour cells was increased after VEGF disrupted the glycocalyx on the surface of vascular endothelial cells (Xia et al., 2022; Zeng et al., 2022). Heparan-sulfate proteoglycans (HPSE) digestion-generated heparan sulfate fragments of about 10 kDa in B16B15b melanoma cell stimulated melanoma migration and angiogenesis, which were possibly due to signaling by some other unknown heparin-binding growth factor(s) (Roy and Marchetti, 2009).

TECs can transform tumour cells into more tumourigenic, invasive and chemoresistant cells (Cao et al., 2017; Hoarau-Véchot et al., 2022). In a bio-3D printed *in vitro* vascular model of neuroblastoma, the invasive behaviour of neuroblastoma cells co-cultured with human umbilical vein endothelial cells was significantly increased (Ning et al., 2022). By inhibiting the expression of lectin-like oxidised LDL receptor 1 (LOX-1) in TECs, the LOX-1 enahnced neutrophil migration was inhibited, and thus the formation of high metastatic-tumor microenvironment was inhibited and lung metastasis of tumour cells can be suppressed (Tsumita et al., 2022).

In addition, TECs can accelerate lung metastasis of tumour cells with low metastatic potential by releasing the vascular secretory factor biglycan (Maishi et al., 2016). TECs express high levels of adhesion molecules and biglycan, which allow vascular endothelial cells to bind to tumour cells, protecting them from circulating anoikis and promoting their metastasis to distant organs (Yadav et al., 2015).

## 5 TECs in anti-angiogenic therapy

Anti-angiogenic drugs are currently used in a combination therapeutic model with immunotherapy and radiotherapy, which demonstrated promising anti-tumor activity in various advanced solid tumors (Shen et al., 2023). Anti-angiogenic therapies alter the immunologically permissive microenvironment of the tumour by reducing the number of regulatory T cells and myeloid-derived suppressor cells (Wallin et al., 2016; Hegde et al., 2018). However, anti-VEGFA therapy is a direct reduction in the rate of tumour growth rather than an induction of tumour regression, and some drugs slow tumour growth without necessarily causing tumour shrinkage (Bagri et al., 2010). In patients with advanced EGFR-mutated non-small cell lung cancer, longer but not significant progression-free survival (PFS) was observed in the erlotinib plus bevacizumab compared with the erlotinib alone (Lee Y. et al., 2023).

Despite the benefits, anti-angiogenic therapies face challenges. Complications and adverse events such as hypertension, hand-foot syndrome, proteinuria and thyroid dysfunction can occur due to the adverse effect of anti-angiogenic drugs on normal blood vessels, as well as the possibility of drug resistance, which often requires drug replacement (Li et al., 2022; Yuan et al., 2022; Lee N. et al., 2023).

Long-term anti-angiogenic therapy induces tumour hypoxia and invasiveness (Pàez-Ribes et al., 2009). Hypoxia also induces tumour angiogenesis through the recruitment of bone marrow-derived endothelial progenitor cells (Gao et al., 2009). Recurrence and metastasis after initial tumour response have also been reported with anti-angiogenic therapy (Mancuso et al., 2006; Allegra et al., 2009; Ebos et al., 2009; Toda et al., 2021). Exosomes generated by TECs in response to anti-angiogenic drugs may also promote tumour cell vascular mimicry and the development of aberrant tumour vasculature (Zeng et al., 2019; Zeng and Fu, 2020). This provides *in vitro* evidence and possible mechanisms by which anti-angiogenic therapy may promote metastasis.

## 6 Conclusion and prospects

**FIGURE 1:**
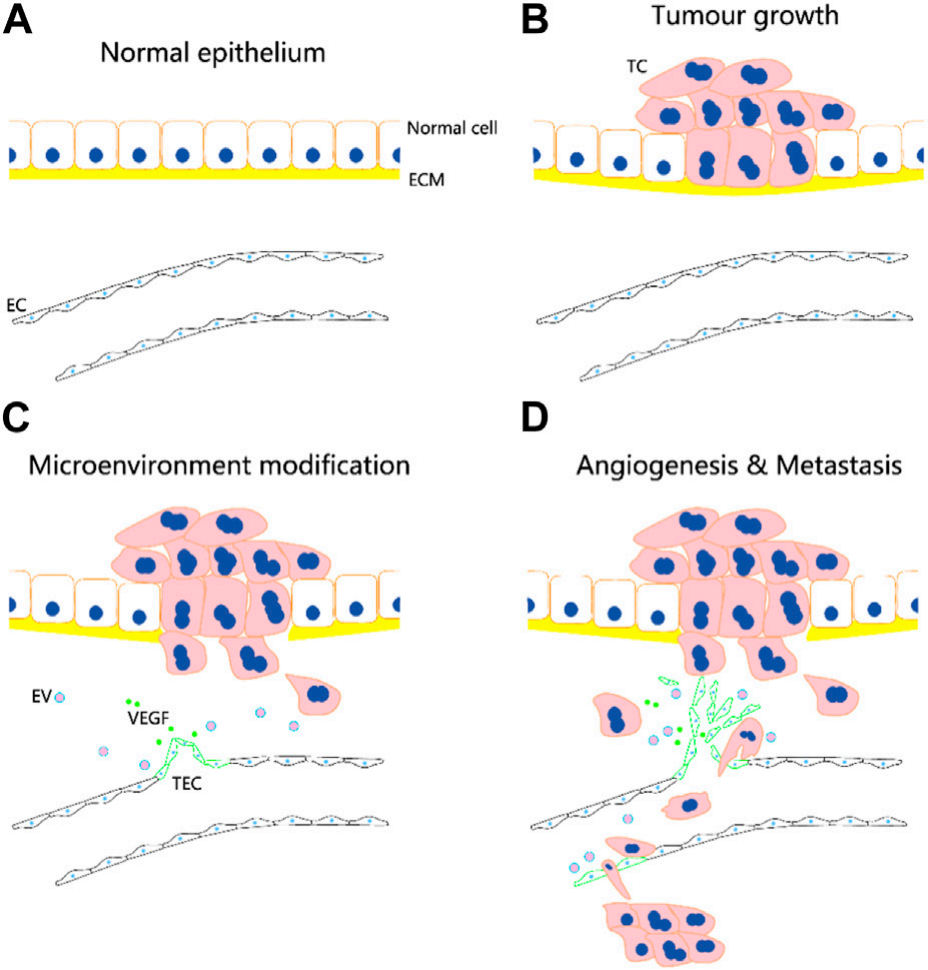
Potential role of tumour-associated endothelial cells (TECs) in tumour angiogenesis and metastasis. TECs are mainly derived from tumour vasculature. They may be formed by uptake of extracellular vesicles (EVs) or non-coding RNAs from tumour cells (TCs) and may undergo transdifferentiation from tumour stem cells and endothelial progenitor cells. **(A–D)** Following the occurrence of tumor, the normal vascular endothelial cells (ECs) are transformed into TECs, which facilitate the infiltration of tumor cells into microcirculation and metastasis through bloodstream. The circulation tumor cells may induce the transformation of endothelial cells in distal vessels into TECs, and aid to their adhesion and invasion for metastatic foci formation.

TECs are mainly derived from tumour vasculature (Figure 1). They may be formed by uptake of extracellular vesicles or non-coding RNAs from tumour cells and may undergo transdifferentiation from tumour stem cells and endothelial progenitor cells, although the exact mechanism of transformation requires further investigation. The formation of TECs may involve fusion between transformed cells and normal stem cells.

**FIGURE 2:**
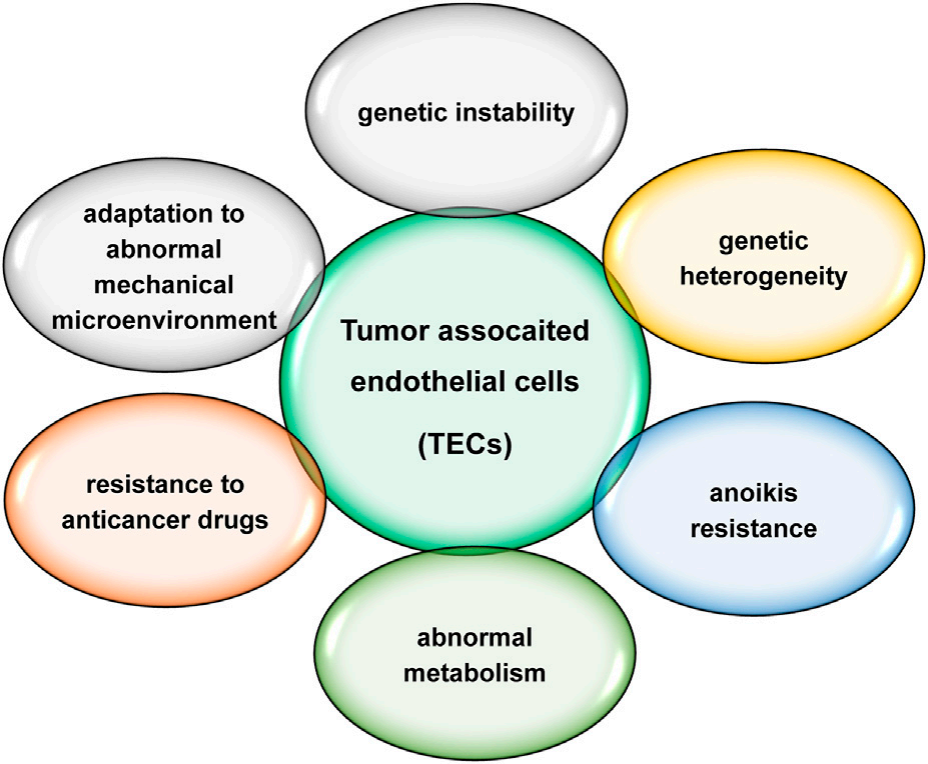
Major characteristics of TECs.

TECs are distinctly different from normal endothelial cells, characterized by genetic instability, genetic heterogeneity, anoikis resistance, abnormal metabolism, resistance to anticancer drugs and adaptation to an abnormal mechanical microenvironment (Figure 2). TECs play an important role in tumour progression and metastasis. Increased evidences suggest that TECs are more promising targets than normal endothelial cells. Circulating TECs may be mesenchymal TECs shed from the neoplastic vasculature (McGuire et al., 2012; Lin, 2020). Circulating TECs reduction after anti-angiogenic therapy and surgical tumour resection (Mehran et al., 2014; Zhang et al., 2021) suggests that circulating TECs should be more specific for cancer and more suitable for prognosis, but it is unclear which precise markers is specific for TECs and appropriate for clinical application.

Furthermore, the complexity of the tumour microenvironment and its intricate details remain unclear, and there is a lack of uniform standards for the detection of TECs. A comprehensive description of the tumour microenvironment and a better understanding of the mechanism underlying how TECs form and function will facilitate the design of novel approaches for overcoming resistance and monitoring the efficacy of anti-angiogenic therapy.

## References

[B1] Aird W. C. (2009). Molecular heterogeneity of tumor endothelium. Cell Tissue Res. 335, 271–281. 10.1007/s00441-008-0672-y 18726119

[B2] Aizarani N. Saviano A. Sagar Mailly L. Durand S. Herman J. S. (2019). A human liver cell atlas reveals heterogeneity and epithelial progenitors. Nature 572, 199–204. 10.1038/s41586-019-1373-2 31292543 PMC6687507

[B3] Akino T. Hida K. Hida Y. Tsuchiya K. Freedman D. Muraki C. (2009). Cytogenetic abnormalities of tumor-associated endothelial cells in human malignant tumors. Am. J. Pathol. 175, 2657–2667. 10.2353/ajpath.2009.090202 19875502 PMC2789618

[B4] Akiyama K. Ohga N. Hida Y. Kawamoto T. Sadamoto Y. Ishikawa S. (2012). Tumor endothelial cells acquire drug resistance by MDR1 up-regulation via VEGF signaling in tumor microenvironment. Am. J. Pathol. 180, 1283–1293. 10.1016/j.ajpath.2011.11.029 22245726

[B5] Allegra C. J. Yothers G. O'Connell M. J. Sharif S. Colangelo L. H. Lopa S. H. (2009). Initial safety report of nsabp C-08: A randomized phase III study of modified FOLFOX6 with or without bevacizumab for the adjuvant treatment of patients with stage II or III colon cancer. J. Clin. Oncol. 27, 3385–3390. 10.1200/JCO.2009.21.9220 19414665 PMC2717026

[B6] Annan D. A. Maishi N. Soga T. Dawood R. Li C. Kikuchi H. (2019). Carbonic anhydrase 2 (CAII) supports tumor blood endothelial cell survival under lactic acidosis in the tumor microenvironment. Cell Commun. Signal 17, 169. 10.1186/s12964-019-0478-4 31847904 PMC6918655

[B7] Bagri A. Berry L. Gunter B. Singh M. Kasman I. Damico L. A. (2010). Effects of anti-VEGF treatment duration on tumor growth, tumor regrowth, and treatment efficacy. Clin. Cancer Res. 16, 3887–3900. 10.1158/1078-0432.CCR-09-3100 20554752

[B8] Balan M. Trusohamn M. Ning F. C. Jacob S. Pietras K. Eriksson U. (2019). Noninvasive intravital high-resolution imaging of pancreatic neuroendocrine tumours. Sci. Rep. 9, 14636. 10.1038/s41598-019-51093-0 31601958 PMC6787246

[B9] Bergsmedh A. Szeles A. Henriksson M. Bratt A. Folkman M. J. Spetz A. L. (2001). Horizontal transfer of oncogenes by uptake of apoptotic bodies. Proc. Natl. Acad. Sci. U. S. A. 98, 6407–6411. 10.1073/pnas.101129998 11353826 PMC33481

[B10] Bussolati B. Deambrosis I. Russo S. Deregibus M. C. Camussi G. (2003). Altered angiogenesis and survival in human tumor-derived endothelial cells. Faseb J. 17, 1159–1161. 10.1096/fj.02-0557fje 12709414

[B11] Butler J. M. Kobayashi H. Rafii S. (2010). Instructive role of the vascular niche in promoting tumour growth and tissue repair by angiocrine factors. Nat. Rev. Cancer 10, 138–146. 10.1038/nrc2791 20094048 PMC2944775

[B12] Cantelmo A. R. Conradi L. C. Brajic A. Goveia J. Kalucka J. Pircher A. (2016). Inhibition of the glycolytic activator PFKFB3 in endothelium induces tumor vessel normalization, impairs metastasis, and improves chemotherapy. Cancer Cell 30, 968–985. 10.1016/j.ccell.2016.10.006 27866851 PMC5675554

[B13] Cao Z. Scandura J. M. Inghirami G. G. Shido K. Ding B. S. Rafii S. (2017). Molecular checkpoint decisions made by subverted vascular niche transform indolent tumor cells into chemoresistant cancer stem cells. Cancer Cell 31, 110–126. 10.1016/j.ccell.2016.11.010 27989801 PMC5497495

[B14] Carlson J. C. Cantu Gutierrez M. Lozzi B. Huang-Hobbs E. Turner W. D. Tepe B. (2021). Identification of diverse tumor endothelial cell populations in malignant glioma. Neuro Oncol. 23, 932–944. 10.1093/neuonc/noaa297 33367832 PMC8168823

[B15] Carmeliet P. Jain R. K. (2000). Angiogenesis in cancer and other diseases. Nature 407, 249–257. 10.1038/35025220 11001068

[B16] Cheng L. Huang Z. Zhou W. Wu Q. Donnola S. Liu J. K. (2013). Glioblastoma stem cells generate vascular pericytes to support vessel function and tumor growth. Cell 153, 139–152. 10.1016/j.cell.2013.02.021 23540695 PMC3638263

[B17] Delia D. Lampugnani M. G. Resnati M. Dejana E. Aiello A. Fontanella E. (1993). CD34 expression is regulated reciprocally with adhesion molecules in vascular endothelial cells *in vitro* . Blood 81, 1001–1008. 10.1182/blood.v81.4.1001.bloodjournal8141001 7679004

[B18] Denzer L. Muranyi W. Schroten H. Schwerk C. (2023). The role of PLVAP in endothelial cells. Cell Tissue Res. 392, 393–412. 10.1007/s00441-023-03741-1 36781482 PMC10172233

[B19] Ebos J. M. Lee C. R. Cruz-Munoz W. Bjarnason G. A. Christensen J. G. Kerbel R. S. (2009). Accelerated metastasis after short-term treatment with a potent inhibitor of tumor angiogenesis. Cancer Cell 15, 232–239. 10.1016/j.ccr.2009.01.021 19249681 PMC4540346

[B20] Ehnfors J. Kost-Alimova M. Persson N. L. Bergsmedh A. Castro J. Levchenko-Tegnebratt T. (2009). Horizontal transfer of tumor DNA to endothelial cells *in vivo* . Cell Death Differ. 16, 749–757. 10.1038/cdd.2009.7 19219067

[B21] Finger E. B. Puri K. D. Alon R. Lawrence M. B. von Andrian U. H. Springer T. A. (1996). Adhesion through L-selectin requires a threshold hydrodynamic shear. Nature 379, 266–269. 10.1038/379266a0 8538793

[B22] Gao D. Nolan D. McDonnell K. Vahdat L. Benezra R. Altorki N. (2009). Bone marrow-derived endothelial progenitor cells contribute to the angiogenic switch in tumor growth and metastatic progression. Biochim. Biophys. Acta 1796, 33–40. 10.1016/j.bbcan.2009.05.001 19460418 PMC3649840

[B23] Glinsky G. V. (2005). Death-from-cancer signatures and stem cell contribution to metastatic cancer. Cell Cycle 4, 1171–1175. 10.4161/cc.4.9.2001 16082216

[B24] Goveia J. Rohlenova K. Taverna F. Treps L. Conradi L. C. Pircher A. (2020). An integrated gene expression landscape profiling approach to identify lung tumor endothelial cell heterogeneity and angiogenic candidates. Cancer Cell 37, 421–436.e13. 10.1016/j.ccell.2020.03.002 32183954

[B25] Guarino B. Katari V. Adapala R. Bhavnani N. Dougherty J. Khan M. (2021). Tumor-derived extracellular vesicles induce abnormal angiogenesis via TRPV4 downregulation and subsequent activation of YAP and VEGFR2. Front. Bioeng. Biotechnol. 9, 790489. 10.3389/fbioe.2021.790489 35004649 PMC8733651

[B26] Hashemi G. Dight J. Khosrotehrani K. Sormani L. (2022). Melanoma tumour vascularization and tissue-resident endothelial progenitor cells. Cancers (Basel) 14, 4216. 10.3390/cancers14174216 36077754 PMC9454996

[B27] He H. Niu C. S. Li M. W. (2012). Correlation between glioblastoma stem-like cells and tumor vascularization. Oncol. Rep. 27, 45–50. 10.3892/or.2011.1484 21971709

[B28] Hegde P. S. Wallin J. J. Mancao C. (2018). Predictive markers of anti-VEGF and emerging role of angiogenesis inhibitors as immunotherapeutics. Semin. Cancer Biol. 52, 117–124. 10.1016/j.semcancer.2017.12.002 29229461

[B29] Hennigs J. K. Matuszcak C. Trepel M. Körbelin J. (2021). Vascular endothelial cells: Heterogeneity and targeting approaches. Cells 10, 2712. 10.3390/cells10102712 34685692 PMC8534745

[B30] Hida K. Hida Y. Amin D. N. Flint A. F. Panigrahy D. Morton C. C. (2004). Tumor-associated endothelial cells with cytogenetic abnormalities. Cancer Res. 64, 8249–8255. 10.1158/0008-5472.CAN-04-1567 15548691

[B31] Hida K. Kawamoto T. Maishi N. Morimoto M. Akiyama K. Ohga N. (2017). miR-145 promoted anoikis resistance in tumor endothelial cells. J. Biochem. 162, 81–84. 10.1093/jb/mvx033 28510655

[B32] Hillen F. Griffioen A. W. (2007). Tumour vascularization: Sprouting angiogenesis and beyond. Cancer Metastasis Rev. 26, 489–502. 10.1007/s10555-007-9094-7 17717633 PMC2797856

[B33] Hoarau-Véchot J. Blot-Dupin M. Pauly L. Touboul C. Rafii S. Rafii A. (2022). Akt-activated endothelium increases cancer cell proliferation and resistance to treatment in ovarian cancer cell organoids. Int. J. Mol. Sci. 23, 14173. 10.3390/ijms232214173 36430649 PMC9694384

[B34] Kikuchi H. Maishi N. Annan D. A. Alam M. T. Dawood R. I. H. Sato M. (2020). Chemotherapy-induced IL8 upregulates MDR1/ABCB1 in tumor blood vessels and results in unfavorable outcome. Cancer Res. 80, 2996–3008. 10.1158/0008-5472.CAN-19-3791 32536602

[B35] Kondoh M. Ohga N. Akiyama K. Hida Y. Maishi N. Towfik A. M. (2013). Hypoxia-induced reactive oxygen species cause chromosomal abnormalities in endothelial cells in the tumor microenvironment. PLoS One 8, e80349. 10.1371/journal.pone.0080349 24260373 PMC3829944

[B36] Lee N. Lee J. L. Lee J. Y. (2023a). Analysis of anti-angiogenesis-related adverse events associated with vascular endothelial growth factor receptor-tyrosine kinase inhibitors (VEGFR-TKIs) in patients with metastatic renal cell carcinoma. Target Oncol. 18, 247–255. 10.1007/s11523-023-00951-z 36826462

[B37] Lee Y. Kim H. R. Hong M. H. Lee K. H. Park K. U. Lee G. K. (2023b). A randomized Phase 2 study to compare erlotinib with or without bevacizumab in previously untreated patients with advanced non-small cell lung cancer with EGFR mutation. Cancer 129, 405–414. 10.1002/cncr.34553 36451343 PMC10100207

[B38] Li D. D. Tao Z. H. Wang B. Y. Wang L. P. Cao J. Hu X. C. (2022). Apatinib plus vinorelbine versus vinorelbine for metastatic triple-negative breast cancer who failed first/second-line treatment: The NAN trial. NPJ Breast Cancer 8, 110. 10.1038/s41523-022-00462-6 36127351 PMC9489776

[B39] Li D. Zhang Z. Xia C. Niu C. Zhou W. (2021). Non-coding RNAs in glioma microenvironment and angiogenesis. Front. Mol. Neurosci. 14, 763610. 10.3389/fnmol.2021.763610 34803608 PMC8595242

[B40] Lin P. P. (2020). Aneuploid circulating tumor-derived endothelial cell (ctec): A novel versatile player in tumor neovascularization and cancer metastasis. Cells 9, 1539. 10.3390/cells9061539 32599893 PMC7349247

[B41] Lin P. P. Gires O. Wang D. D. Li L. Wang H. (2017). Comprehensive *in situ* co-detection of aneuploid circulating endothelial and tumor cells. Sci. Rep. 7, 9789. 10.1038/s41598-017-10763-7 28852197 PMC5575124

[B42] Liu W. Chen Y. Xu W. Wang W. Tang L. Xia R. (2020). Fentanyl stimulates tumor angiogenesis via activating multiple pro-angiogenic signaling pathways. Biochem. Biophys. Res. Commun. 532, 225–230. 10.1016/j.bbrc.2020.08.038 32861420

[B43] Lu X. Baudouin S. V. Gillespie J. I. Anderson J. J. Dickinson A. M. (2007). A comparison of CFU-GM, BFU-E and endothelial progenitor cells using *ex vivo* expansion of selected cord blood CD133(+) and CD34(+) cells. Cytotherapy 9, 292–300. 10.1080/14653240701247853 17464761

[B44] Maddison K. Bowden N. A. Graves M. C. Tooney P. A. (2023). Characteristics of vasculogenic mimicry and tumour to endothelial transdifferentiation in human glioblastoma: A systematic review. BMC Cancer 23, 185. 10.1186/s12885-023-10659-y 36823554 PMC9948311

[B45] Maishi N. Ohba Y. Akiyama K. Ohga N. Hamada J. Nagao-Kitamoto H. (2016). Tumour endothelial cells in high metastatic tumours promote metastasis via epigenetic dysregulation of biglycan. Sci. Rep. 6, 28039. 10.1038/srep28039 27295191 PMC4904795

[B46] Mancuso M. R. Davis R. Norberg S. M. O'Brien S. Sennino B. Nakahara T. (2006). Rapid vascular regrowth in tumors after reversal of VEGF inhibition. J. Clin. Invest. 116, 2610–2621. 10.1172/JCI24612 17016557 PMC1578604

[B47] Matsuda K. Ohga N. Hida Y. Muraki C. Tsuchiya K. Kurosu T. (2010). Isolated tumor endothelial cells maintain specific character during long-term culture. Biochem. Biophys. Res. Commun. 394, 947–954. 10.1016/j.bbrc.2010.03.089 20302845

[B48] McGuire T. F. Sajithlal G. B. Lu J. Nicholls R. D. Prochownik E. V. (2012). *In vivo* evolution of tumor-derived endothelial cells. PLoS One 7, e37138. 10.1371/journal.pone.0037138 22623986 PMC3356387

[B49] Mehran R. Nilsson M. Khajavi M. Du Z. Cascone T. Wu H. K. (2014). Tumor endothelial markers define novel subsets of cancer-specific circulating endothelial cells associated with antitumor efficacy. Cancer Res. 74, 2731–2741. 10.1158/0008-5472.CAN-13-2044 24626092 PMC4024326

[B50] Morikawa S. Baluk P. Kaidoh T. Haskell A. Jain R. K. McDonald D. M. (2002). Abnormalities in pericytes on blood vessels and endothelial sprouts in tumors. Am. J. Pathol. 160, 985–1000. 10.1016/S0002-9440(10)64920-6 11891196 PMC1867175

[B51] Motzer R. J. Taylor M. H. Evans T. R. J. Okusaka T. Glen H. Lubiniecki G. M. (2022). Lenvatinib dose, efficacy, and safety in the treatment of multiple malignancies. Expert Rev. Anticancer Ther. 22, 383–400. 10.1080/14737140.2022.2039123 35260027 PMC9484451

[B52] Naschberger E. Schellerer V. S. Rau T. T. Croner R. S. Stürzl M. (2011). Isolation of endothelial cells from human tumors. Methods Mol. Biol. 731, 209–218. 10.1007/978-1-61779-080-5_18 21516410

[B53] Neve Polimeno M. Ierano C. D'Alterio C. Simona Losito N. Napolitano M. Portella L. (2015). CXCR4 expression affects overall survival of HCC patients whereas CXCR7 expression does not. Cell Mol. Immunol. 12, 474–482. 10.1038/cmi.2014.102 25363530 PMC4496532

[B54] Ning L. Shim J. Tomov M. L. Liu R. Mehta R. Mingee A. (2022). A 3D bioprinted *in vitro* model of neuroblastoma recapitulates dynamic tumor-endothelial cell interactions contributing to solid tumor aggressive behavior. Adv. Sci. (Weinh) 9, e2200244. 10.1002/advs.202200244 35644929 PMC9376856

[B55] Ohga N. Ishikawa S. Maishi N. Akiyama K. Hida Y. Kawamoto T. (2012). Heterogeneity of tumor endothelial cells: Comparison between tumor endothelial cells isolated from high- and low-metastatic tumors. Am. J. Pathol. 180, 1294–1307. 10.1016/j.ajpath.2011.11.035 22245217

[B56] Oka C. Miyake Y. Tateishi K. Kawabata Y. Iwashita H. Yamamoto T. (2023). Thigh leiomyosarcoma-derived brain metastasis with intracerebral hematoma: A case report and literature review. Surg. Neurol. Int. 14, 80. 10.25259/SNI_113_2023 37025533 PMC10070302

[B57] Onken M. D. Mooren O. L. Mukherjee S. Shahan S. T. Li J. Cooper J. A. (2014). Endothelial monolayers and transendothelial migration depend on mechanical properties of the substrate. Cytoskelet. Hob. 71, 695–706. 10.1002/cm.21203 PMC4373417 25545622

[B58] Onyeisi J. O. S. Pernambuco Filho P. C. A. Mesquita A. P. S. Azevedo L. C. Nader H. B. Lopes C. C. (2020). Effects of syndecan-4 gene silencing by micro RNA interference in anoikis resistant endothelial cells: Syndecan-4 silencing and anoikis resistance. Int. J. Biochem. Cell Biol. 128, 105848. 10.1016/j.biocel.2020.105848 32927086

[B59] Otsubo T. Hida Y. Ohga N. Sato H. Kai T. Matsuki Y. (2014). Identification of novel targets for antiangiogenic therapy by comparing the gene expressions of tumor and normal endothelial cells. Cancer Sci. 105, 560–567. 10.1111/cas.12394 24602018 PMC4317838

[B60] Pàez-Ribes M. Allen E. Hudock J. Takeda T. Okuyama H. Viñals F. (2009). Antiangiogenic therapy elicits malignant progression of tumors to increased local invasion and distant metastasis. Cancer Cell 15, 220–231. 10.1016/j.ccr.2009.01.027 19249680 PMC2874829

[B61] Paszek M. J. Zahir N. Johnson K. R. Lakins J. N. Rozenberg G. I. Gefen A. (2005). Tensional homeostasis and the malignant phenotype. Cancer Cell 8, 241–254. 10.1016/j.ccr.2005.08.010 16169468

[B62] Patel J. Seppanen E. J. Rodero M. P. Wong H. Y. Donovan P. Neufeld Z. (2017). Functional definition of progenitors versus mature endothelial cells reveals key SoxF-dependent differentiation process. Circulation 135, 786–805. 10.1161/CIRCULATIONAHA.116.024754 27899395

[B63] Pyaskovskaya O. N. Kolesnik D. L. Garmanchouk L. V. Yanish Yu V. Solyanik G. I. (2021). Role of tumor/endothelial cell interactions in tumor growth and metastasis. Exp. Oncol. 43, 104–110. 10.32471/exp-oncology.2312-8852.vol-43-no-2.16157 34190519

[B64] Qi Y. Song Y. Cai M. Li J. Yu Z. Li Y. (2023). Vascular endothelial growth factor A is a potential prognostic biomarker and correlates with immune cell infiltration in hepatocellular carcinoma. J. Cell Mol. Med. 27, 538–552. 10.1111/jcmm.17678 36729917 PMC9930434

[B65] Quirici N. Soligo D. Caneva L. Servida F. Bossolasco P. Deliliers G. L. (2001). Differentiation and expansion of endothelial cells from human bone marrow CD133(+) cells. Br. J. Haematol. 115, 186–194. 10.1046/j.1365-2141.2001.03077.x 11722432

[B66] Rantakari P. Auvinen K. Jäppinen N. Kapraali M. Valtonen J. Karikoski M. (2015). The endothelial protein PLVAP in lymphatics controls the entry of lymphocytes and antigens into lymph nodes. Nat. Immunol. 16, 386–396. 10.1038/ni.3101 25665101

[B67] Ren B. Rose J. B. Liu Y. Jaskular-Sztul R. Contreras C. Beck A. (2019). Heterogeneity of vascular endothelial cells, de novo arteriogenesis and therapeutic implications in pancreatic neuroendocrine tumors. J. Clin. Med. 8, 1980. 10.3390/jcm8111980 31739580 PMC6912347

[B68] Ribatti D. (2008). Judah Folkman, a pioneer in the study of angiogenesis. Angiogenesis 11, 3–10. 10.1007/s10456-008-9092-6 18247146 PMC2268723

[B69] Ricci-Vitiani L. Pallini R. Biffoni M. Todaro M. Invernici G. Cenci T. (2010). Tumour vascularization via endothelial differentiation of glioblastoma stem-like cells. Nature 468, 824–828. 10.1038/nature09557 21102434

[B70] Riddle R. B. Jennbacken K. Hansson K. M. Harper M. T. (2022). Endothelial inflammation and neutrophil transmigration are modulated by extracellular matrix composition in an inflammation-on-a-chip model. Sci. Rep. 12, 6855. 10.1038/s41598-022-10849-x 35477984 PMC9046410

[B71] Rodriguez F. J. Orr B. A. Ligon K. L. Eberhart C. G. (2012). Neoplastic cells are a rare component in human glioblastoma microvasculature. Oncotarget 3, 98–106. 10.18632/oncotarget.427 22298889 PMC3292896

[B72] Roy M. Marchetti D. (2009). Cell surface heparan sulfate released by heparanase promotes melanoma cell migration and angiogenesis. J. Cell Biochem. 106, 200–209. 10.1002/jcb.22005 19115257 PMC2736788

[B73] Sellner F. Thalhammer S. Klimpfinger M. (2022). Isolated pancreatic metastases of renal cell cancer: Genetics and epigenetics of an unusual tumour entity. Cancers (Basel) 14, 1539. 10.3390/cancers14061539 35326690 PMC8945920

[B74] Shen J. Yan J. Du J. Li X. Wei J. Liu Q. (2023). Multicenter, single-arm, phase II study (CAP) of radiotherapy plus liposomal irinotecan followed by camrelizumab and anti-angiogenic treatment in advanced solid tumors. Front. Immunol. 14, 1133689. 10.3389/fimmu.2023.1133689 37056765 PMC10086408

[B75] Shenoy A. K. Pi L. Ligocki A. P. Hosaka K. Cogle C. R. Scott E. W. (2023). Targeting redundant ROBO1 and SDF-1 pathways prevents adult hemangioblast derived-EPC and CEC activity effectively blocking tumor neovascularization. Stem Cell Rev. Rep. 19, 928–941. 10.1007/s12015-022-10498-7 36652143

[B76] Shi T. Zhang C. Xia S. (2022). The potential roles and mechanisms of non-coding RNAs in cancer anoikis resistance. Mol. Cell Biochem. 477, 1371–1380. 10.1007/s11010-022-04384-6 35142950

[B77] St Croix B. Rago C. Velculescu V. Traverso G. Romans K. E. Montgomery E. (2000). Genes expressed in human tumor endothelium. Science 289, 1197–1202. 10.1126/science.289.5482.1197 10947988

[B78] Stacer A. C. Fenner J. Cavnar S. P. Xiao A. Zhao S. Chang S. L. (2016). Endothelial CXCR7 regulates breast cancer metastasis. Oncogene 35, 1716–1724. 10.1038/onc.2015.236 26119946 PMC4486335

[B79] Streubel B. Chott A. Huber D. Exner M. Jäger U. Wagner O. (2004). Lymphoma-specific genetic aberrations in microvascular endothelial cells in B-cell lymphomas. N. Engl. J. Med. 351, 250–259. 10.1056/NEJMoa033153 15254283

[B80] Tao Z. Huang C. Wang D. Wang Q. Gao Q. Zhang H. (2023). Lactate induced mesenchymal stem cells activation promotes gastric cancer cells migration and proliferation. Exp. Cell Res. 424, 113492. 10.1016/j.yexcr.2023.113492 36702194

[B81] Toda S. Iwasaki H. Murayama D. Nakayama H. Suganuma N. Masudo K. (2021). Invasive procedures in patients undergoing treatment with lenvatinib for thyroid cancer. Mol. Clin. Oncol. 14, 81. 10.3892/mco.2021.2243 33758662 PMC7947953

[B82] Tsumita T. Maishi N. Annan D. A. Towfik M. A. Matsuda A. Onodera Y. (2022). The oxidized-LDL/LOX-1 axis in tumor endothelial cells enhances metastasis by recruiting neutrophils and cancer cells. Int. J. Cancer 151, 944–956. 10.1002/ijc.34134 35608341

[B83] Wallin J. J. Bendell J. C. Funke R. Sznol M. Korski K. Jones S. (2016). Atezolizumab in combination with bevacizumab enhances antigen-specific T-cell migration in metastatic renal cell carcinoma. Nat. Commun. 7, 12624. 10.1038/ncomms12624 27571927 PMC5013615

[B84] Wang R. Chadalavada K. Wilshire J. Kowalik U. Hovinga K. E. Geber A. (2010). Glioblastoma stem-like cells give rise to tumour endothelium. Nature 468, 829–833. 10.1038/nature09624 21102433

[B85] Wang R. Zhong H. Wang C. Huang X. Huang A. Du N. (2023). Tumor malignancy by genetic transfer between cells forming cell-in-cell structures. Cell Death Dis. 14, 195. 10.1038/s41419-023-05707-1 36914619 PMC10011543

[B86] Wang Z. Zhao K. Hackert T. Zöller M. (2018). CD44/CD44v6 a reliable companion in cancer-initiating cell maintenance and tumor progression. Front. Cell Dev. Biol. 6, 97. 10.3389/fcell.2018.00097 30211160 PMC6122270

[B87] Wu W. Wang X. Liao L. Chen J. Wang Y. Yao M. (2023). The TRPM7 channel reprograms cellular glycolysis to drive tumorigenesis and angiogenesis. Cell Death Dis. 14, 183. 10.1038/s41419-023-05701-7 36878949 PMC9988972

[B88] Wu X. Li F. Wang X. Li C. Meng Q. Wang C. (2018). Antibiotic bedaquiline effectively targets growth, survival and tumor angiogenesis of lung cancer through suppressing energy metabolism. Biochem. Biophys. Res. Commun. 495, 267–272. 10.1016/j.bbrc.2017.10.136 29107691

[B89] Xia Y. Li Y. Fu B. M. (2022). Differential effects of vascular endothelial growth factor on glycocalyx of endothelial and tumor cells and potential targets for tumor metastasis. Apl. Bioeng. 6, 016101. 10.1063/5.0064381 35071967 PMC8769769

[B90] Xiong Y. Q. Sun H. C. Zhang W. Zhu X. D. Zhuang P. Y. Zhang J. B. (2009). Human hepatocellular carcinoma tumor-derived endothelial cells manifest increased angiogenesis capability and drug resistance compared with normal endothelial cells. Clin. Cancer Res. 15, 4838–4846. 10.1158/1078-0432.CCR-08-2780 19638466

[B91] Yadav A. Kumar B. Yu J. G. Old M. Teknos T. N. Kumar P. (2015). Tumor-associated endothelial cells promote tumor metastasis by chaperoning circulating tumor cells and protecting them from anoikis. PLoS One 10, e0141602. 10.1371/journal.pone.0141602 26509633 PMC4624958

[B92] Yuan S. Fu Q. Zhao L. Fu X. Li T. Han L. (2022). Efficacy and safety of apatinib in patients with recurrent or refractory melanoma. Oncologist 27, e463–e470. 10.1093/oncolo/oyab068 35348754 PMC9177116

[B93] Zabel B. A. Wang Y. Lewén S. Berahovich R. D. Penfold M. E. Zhang P. (2009). Elucidation of CXCR7-mediated signaling events and inhibition of CXCR4-mediated tumor cell transendothelial migration by CXCR7 ligands. J. Immunol. 183, 3204–3211. 10.4049/jimmunol.0900269 19641136

[B94] Zeng Y. Fu B. M. (2020). Resistance mechanisms of anti-angiogenic therapy and exosomes-mediated revascularization in cancer. Front. Cell Dev. Biol. 8, 610661. 10.3389/fcell.2020.610661 33363174 PMC7755714

[B95] Zeng Y. Qiu Y. Jiang W. Fu B. M. (2022). Glycocalyx acts as a central player in the development of tumor microenvironment by extracellular vesicles for angiogenesis and metastasis. Cancers (Basel) 14, 5415. 10.3390/cancers14215415 36358833 PMC9655334

[B96] Zeng Y. Yao X. Liu X. He X. Li L. Liu X. (2019). Anti-angiogenesis triggers exosomes release from endothelial cells to promote tumor vasculogenesis. J. Extracell. Vesicles 8, 1629865. 10.1080/20013078.2019.1629865 31258881 PMC6586113

[B97] Zhang L. Li S. Li L. Chen Z. Yang Y. (2018). COX-2 inhibition in the endothelium induces glucose metabolism normalization and impairs tumor progression. Mol. Med. Rep. 17, 2937–2944. 10.3892/mmr.2017.8270 29257333 PMC5783508

[B98] Zhang L. Zhang X. Liu Y. Zhang T. Wang Z. Gu M. (2020). PD-L1(+) aneuploid circulating tumor endothelial cells (CTECs) exhibit resistance to the checkpoint blockade immunotherapy in advanced NSCLC patients. Cancer Lett. 469, 355–366. 10.1016/j.canlet.2019.10.041 31678168

[B99] Zhang T. Zhang L. Gao Y. Wang Y. Liu Y. Zhang H. (2021). Role of aneuploid circulating tumor cells and CD31(+) circulating tumor endothelial cells in predicting and monitoring anti-angiogenic therapy efficacy in advanced NSCLC. Mol. Oncol. 15, 2891–2909. 10.1002/1878-0261.13092 34455700 PMC8564645

[B100] Zhou J. Cheng M. Liao Y. H. Hu Y. Wu M. Wang Q. (2013). Rosuvastatin enhances angiogenesis via eNOS-dependent mobilization of endothelial progenitor cells. PLoS One 8, e63126. 10.1371/journal.pone.0063126 23704894 PMC3660394

